# Digit ratio as an endophenotype in a schizophrenia population

**DOI:** 10.4102/sajpsychiatry.v27i0.1587

**Published:** 2021-03-03

**Authors:** Wilhelm D.B. Nieuwoudt, Inge M. Smit, Dana Niehaus, Liezl Koen, Esme Jordaan

**Affiliations:** 1 Department of Psychiatry, Faculty of Medicine and Health Sciences, Stellenbosch University, Cape Town, South Africa; 2 Biostatistics Unit, South African Medical Research Council, Cape Town, South Africa

**Keywords:** digit ratio, 2D:4D, digits, schizophrenia, endophenotype, population, male, female

## Abstract

**Background:**

Schizophrenia is a debilitating mental health condition affecting the lives of many South Africans. The origins of the heterogeneity in the presentation of the illness remain uncertain.

**Aim:**

This cross-sectional study performed a retrospective data analysis to determine the usefulness of digit ratio as an endophenotype in a South African schizophrenia population.

**Setting:**

A large genetic study in a South African schizophrenia population recruited patients from services in the Western and Eastern Cape.

**Methods:**

Complete clinical histories were captured for participants, including sets of images of the face and extremities. Software was utilised to measure the lengths of participants’ digits from said images and digit ratios (2D:4D) were calculated. Descriptive analyses were performed on the ratios and statistical differences in digit ratio means were calculated between groups characterised by sex, age of onset and the presence vs absence of positive symptoms. Linear modelling was utilised to assess for correlates between 2D:4D and positive and negative symptom severity using scores obtained from the Positive and Negative Syndrome Scale (PANSS) and Scale for the Assessment of Negative Symptoms (SANS).

**Results:**

2D:4D in male participants did not significantly differ from female participants as in healthy populations. 2D:4D did not significantly correlate with the severity of positive or negative symptoms and 2D:4D means between groups did not significantly relate to age of onset.

**Conclusion:**

2D:4D appears to be a possible endophenotype in schizophrenia in this population. 2D:4D, however, may not be as readily identifiable as certain minor physical anomalies and neurological soft signs significantly associated with schizophrenia in this population.

## Background

The lifetime prevalence of mental disorders in South Africans is estimated at 30.8%.^1^ Schizophrenia is a prevalent example of such a disorder affecting the lives of many South Africans and contributes majorly to global disability. Despite the condition presenting with the core features of psychosis, schizophrenia still varies considerably in its manifestations. It remains uncertain whether schizophrenia is a single disorder with a varying clinical picture or in fact a cluster of syndromes which are either overlapping or distinctive in their pathophysiology.^2^

Endophenotypes are defined as measurable factors – unobserved by the unaided eye – that occur intermediately along a path with clinical disease and a distal genotype at either end.^3^ Such factors may be physiological, anatomical, symptomatic or biochemical in nature and suggest genetic underpinnings to disease.^3^ Endophenotypes may therefore be of use in identifying possible subtypes in schizophrenia. Suggested endophenotypes in schizophrenia, which is believed to have a strong genetic aetiological component, include symptomatology, physical characteristics and neurodevelopmental insults.

The ratio of the lengths of the second to fourth fingers is commonly referred to as 2D:4D or ‘digit ratio’. Links between digit ratio and in utero testosterone exposure, body-composition, alcohol dependency, risk-taking, moral judgement, psychopathy and sexual orientation have previously been researched.

Differences in digit ratio, specifically between males and females, have been widely described and are likely thought to be because of the action of androgens during the perinatal period according to Ventura et al.^4^ This normal sexual dimorphism is evidenced by a lower 2D:4D or ‘masculine pattern’ in males where the ring finger typically grows longer in relation to the index (low digit ratio), compared to females with a longer index in relation to the ring finger (high digit ratio) or ‘feminine pattern’. This was most significantly exhibited on the right hand in a meta-analysis by Hönekopp et al.^5^

Authors have previously investigated associations between digit ratio and specific symptoms of schizophrenia. Bolu et al. found that right and left 2D:4D ratios were oppositely affected in a Turkish Caucasian male population with schizophrenia.^6^ Here, participants exhibited higher right-sided and lower left-sided digit ratios than healthy controls.^6^ Upon considering the close temporal relationship between prenatal development of the extremities and the brain, the authors thought the unequal expression of left and right 2D:4D to be partially explained by theories on the influence of in utero sex hormone exposure on cerebral lateralisation.^6^

Cerebral lateralisation is considered a normal developmental phenomenon where the left and right cerebral hemispheres develop in an asymmetrical fashion. Neural structures fulfilling specific functions ‘lateralise’ to either the left or right cerebral hemisphere and have been found to do so more significantly in males, who are normally exposed to higher levels of prenatal testosterone.^7^ This is based on the theory put forth by Geschwind and Galaburda to show the influence of in utero testosterone on cerebral lateralisation.^8^

Venkatasubramanian et al. investigated the connection between abnormal cerebral lateralisation and schizophrenia by comparing 2D:4D of affected participants exhibiting Schneiderian first rank symptoms (FRS) with that of the affected participants who did not as well as healthy controls with digit ratio having been shown to be an indicator of cerebral lateralisation and the presence of FRS being thought to occur in aberrant lateralisation.^9^

These FRSs are listed as auditory hallucinations, thought broadcasting (believing others can hear your thoughts), thought insertion (believing thoughts are being inserted into your mind without your control or consent), thought withdrawal (believing thoughts are being removed from your mind) and delusions.^10^ The presence of FRS was found to correlate with a lower left-sided 2D:4D compared to healthy controls, supporting the association between aberrant cerebral lateralisation and the development of schizophrenia.^9,10^ Importantly, FRSs are not synonymous with the current concept of positive symptoms in schizophrenia, but encapsulate the core features of active psychosis and therefore remain useful for reference and comparison.

More recently, Paipa et al. suggested a protective effect of higher in utero exposure of females to oestrogen (high 2D:4D) and males to testosterone (low 2D:4D) from developing negative symptoms, but a predisposing effect of said exposures to developing affective symptoms, such as depression in schizophrenia.^11^

In a Chinese population with schizophrenia, 2D:4D ratios in both male and female participants were found to be significantly higher than in matched controls. A significant difference in right-sided 2D:4D between male patients and controls was found as opposed to a difference in both left and right-sided 2D:4D between female patients and controls. The authors partially interpreted the sex difference as a manifestation of the effect of testosterone on cerebral lateralisation as cited by Qian et al.^12^ In this population, male 2D:4D showed weak inverse proportionality to age of onset, showing possible potential for 2D:4D as an early indicator of schizophrenia in males.^12^

Our study attempts a unique analysis within a genetically homogenous South African population and attempts to expand on local data whilst further establishing universality of findings in a global context.

## Aim

The aim of this cross-sectional study is to determine the utility of digit ratio as an endophenotype in a South African schizophrenia population by performing a retrospective data analysis. It is the wish of the researchers that this study benefits the ongoing search for clarity regarding the nature and development of schizophrenia.

## Methods

The population investigated for the retrospective analysis was derived from a large study by Niehaus et al.^13^ This population was defined originally as consenting individuals known with schizophrenia of Xhosa heritage, recruited from in-patient hospital services and community clinics throughout the Western and Eastern Cape provinces of South Africa. Complete psychiatric histories were obtained from participants, which include information on current and previous illness, total number and duration of psychotic episodes and hospitalisations, past and present treatment, age of onset, suicide risk and co-morbidities.

Gathered information included a standardised set of digital images of the faces, hands and feet of participants. These data and images of participants’ hands were utilised for *n* = 184 participants specifically recruited from the Western Cape Metropole to perform the current study. The population consisted of 152 males and 32 females with ages ranging from 17 to 66 years (mean = 35, 36 years, SD = 11, 27).

Image measuring software was used to accurately measure – in length − the second (2D) and fourth (4D) digits on the palmar aspects of both hands of participants from the most proximal basal creases where fingers join the hands to the very tips. The application ‘ImageJ’ allowed the measurer to place fine points at the bases and tips of fingers on the images and then reported the distance in pixels. An added zoom function aided in greater accuracy regarding point placement for measurement. The distance values of 2D and 4D of participants were captured and 2D:4D ratios were calculated from tabulated measurements. 2D:4D thus = distance 2D/distance 4D and was tabulated electronically as the resultant number.

Digit ratios were captured along with clinical parameters, that is, the presence versus the absence of positive symptoms as well as symptom severity, sex and age of onset. Descriptive analyses were performed comparing 2D:4D between groups using Statistical Product and Service Solutions (SPSS) 26.0 statistical software.^14^

General linear modelling was used to test differences in the digit ratio means between groups and test for significant correlations between 2D:4D and symptomatology.

For the assessment of severity, symptom scores of participants were captured for positive and negative symptoms. These scores were calculated as a composite of the positive symptoms on the Positive and Negative Syndrome Scale (PANSS)^15^ to assess positive symptom severity (mean score = 2.730, SD males = 0.315, SD females = 0.675). The modified Scale for the Assessment of Negative Symptoms (SANS)^16^ was utilised to assess negative symptom severity (mean score = 9.494, SD males = 3.567, SD females = 4.269). Linear modelling was then used to assess left and right 2D:4D and its correlation with the PANSS composite and SANS scores of participants. Mean 2D:4D of participants showing an absence of positive symptoms at the time of investigation (*n* = 116) was compared to that of participants who displayed positive symptoms (*n* = 68). This was interpreted as a possible indicator of the relationship between symptom predominance and 2D:4D with negative symptoms being present in virtually all participants (*n* = 183).

Age of onset of disease was defined as the age at which the diagnosis of schizophrenia was made in participants. Age of onset of disease in the population ranged from 13 to 51 years (mean = 23.75 years, SD = 6.96). Mean 2D:4D of an earlier onset group of 13–20 years (SD = 2.851) was compared to the uncapped range.

Lastly, 2D:4D means of male and female participants were compared.

### Ethical considerations

Ethical approval to conduct the study was obtained from the Health and Undergraduate Research Ethics Committee of the University of Stellenbosch (reference #U19/07/030).

## Results

The main finding of the analyses performed is that 2D:4D of male participants did not significantly differ from that of female participants (Table 1).

**TABLE 1:** Mean 2D:4D of male and female participants.

R/L	Sex	*N*	Mean	Standard deviation
L2D:4D	female	32	0.9753	0.05113
	male	152	0.9659	0.05021
R2D:4D	female	32	0.9909	0.05778
	male	152	0.9767	0.05668

2D:4D means in participants were not found to differ significantly between individuals displaying only negative symptoms and those displaying positive and negative symptoms. 2D:4D did not correlate significantly with described symptom scores. This finding was repeated upon separate analysis of male and female 2D:4D characterised by symptomatology (Table 2).

**TABLE 2:** Positive and negative symptom scores and their digit ratio correlates.

Symptom domains	Correlates	R2D:4D ratio	L2D:4D ratio
Negative symptoms (SANS scores) (Mean = 9.494)	Pearson correlation	0.040	0.095
	Sig. (2-tailed)	0.592	0.201
	*N*	183	183
Positive symptoms (Composite of positive symptoms in the PANSS) (Mean = 2.730)	Pearson correlation	−0.014	−0.035
	Sig. (2-tailed)	0.859	0.657
	*N*	167	167

SANS, scale for the assessment of negative symptoms; PANSS, positive and negative syndrome scale.

Note: No significant relationship between digit ratio and age of disease onset was demonstrable.

## Discussion

This study explored the potential use of 2D:4D as an endophenotype in a South African schizophrenia population. The lack of difference found between mean 2D:4D of male and female participants and consequent loss of normal sexual dimorphism is a significant finding as differences in 2D:4D between healthy males and females are well described.^5^

These differences are believed to arise from differences in exposure of in utero sex hormone,^4^ which is proposed as a factor influencing neurodevelopment and cerebral lateralisation and is believed to occur to a lesser degree in males with schizophrenia than in their healthy counterparts.^8^

Considering 2D:4D as an endophenotype in the light of the above finding, it is uncertain how closely 2D:4D relates to the genetics underpinning schizophrenia as opposed to being more representative of the sexual development of individuals with schizophrenia, which in turn may add nuance to their presentation.

Regarding the relationship between 2D:4D and clinical parameters in schizophrenia, previous studies have focused mainly on comparison between patients and healthy individuals and consequently consisted of smaller patient samples.

This study attempts to further establish universality of findings and better delineate the heterogeneity of the schizophrenia spectrum by making comparisons within a larger South African schizophrenia patient population.

Of interest to our analysis was whether symptom severity or predominance could be an indicator of further genetically underpinned heterogeneity within the clinical schizophrenia spectrum and whether or not digit ratio could be an indicator of this. For this purpose, patient-patient comparisons were done. Our analyses were not suggestive of such a relationship.

It is possible that genes underpinning clinical heterogeneity are not closely related to 2D:4D as a phenotypical characteristic; besides the clinical heterogeneity in schizophrenia is influenced by an array of other factors – genetics being one – or a combination of the above. This in turn would render 2D:4D an inadequately specific marker of said heterogeneity. It is also possible that pharmacological treatment may have a confounding effect on observations over and above the factors mentioned.

Regarding the age of onset, a relatively young mean was found in our population at roughly 23 years. Here, a disproportionate number of patients who were diagnosed at a younger age could skew the analysis. This may be a reason for not having replicated the 2D:4D or age of onset relationship of Qian et al.^12^ Another reason may be that 2D:4D differentiation does not occur significantly between individuals who develop disease earlier in life and those who do not and that this differentiation is only apparent between healthy individuals and those with schizophrenia.

As a physical attribute or measurement thereof, 2D:4D shows considerable variability within both healthy and patient populations.^11^ This is further evidenced by the bell-curve like distribution of 2D:4D in our participants. Given that 2D:4D group means are further analysed, it is important to remember that 2D:4D may not ostensibly represent a trend or mean associated with pathology when measured in a given individual.

## Limitations

A robustly sized, population-specific group of healthy individuals was not available for the retrospective analysis and investigating healthy individuals in this population remains an opportunity for expansion upon our current findings.

Furthermore, the use of images may have resulted in a slightly greater error margin than direct observation of physical characteristics. The standardised method of imaging, accurate measuring software and larger sample size were deemed adequate to acceptably offset such an error.

## Conclusion

Sexual dimorphism in 2D:4D is well described.^5^ The loss of this sexual dimorphism amongst our population is echoed in other schizophrenia populations with males exhibiting higher digit ratios (longer index finger relative to the ring finger) and more closely matching their female counterparts as opposed to healthy individuals.^12^ This finding in males suggests a possible link between intrauterine testosterone exposure and the development of schizophrenia.

2D:4D ultimately shows promise as an endophenotype in schizophrenia considering its association with the condition across multiple populations and our finding in further support thereof. 2D:4D may, however, be less readily identifiable than other minor physical anomalies^17^ and neurological soft signs^2^ which have also shown significant association with schizophrenia in this population.

## Recommendations

The analysis provides further data for comparison with a robust sample size of healthy individuals in this population for further expansion on its findings.
